# CL-L1 and CL-K1 Exhibit Widespread Tissue Distribution With High and Co-Localized Expression in Secretory Epithelia and Mucosa

**DOI:** 10.3389/fimmu.2018.01757

**Published:** 2018-07-31

**Authors:** Soren W. K. Hansen, Josephine B. Aagaard, Karen B. Bjerrum, Eva K. Hejbøl, Ole Nielsen, Henrik D. Schrøder, Karsten Skjoedt, Anna L. Sørensen, Jonas H. Graversen, Maiken L. Henriksen

**Affiliations:** ^1^Institute of Cancer and Inflammation Research, University of Southern Denmark, Odense, Denmark; ^2^Department of Pathology, Odense University Hospital, Odense, Denmark

**Keywords:** collectin, complement system, 3MC syndrome, mucosal immunology, innate immunity

## Abstract

Collectin liver 1 (CL-L1, alias collectin 10) and collectin kidney 1 (CL-K1, alias collectin 11) are oligomeric pattern recognition molecules associated with the complement system, and mutations in either of their genes may lead to deficiency and developmental defects. The two collectins are reportedly localized and synthesized in the liver, kidneys, and adrenals, and can be found in the circulation as heteromeric complexes (CL-LK), which upon binding to microbial high mannose-like glycoconjugates activates the complement system *via* the lectin activation pathway. The tissue distribution of homo- vs. heteromeric CL-L1 and -K1 complexes, the mechanism of heteromeric complex formation and in which tissues this occurs, is hitherto incompletely described. We have by immunohistochemistry using monoclonal antibodies addressed the precise cellular localization of the two collectins in the main human tissues. We find that the two collectins have widespread and almost identical tissue distribution with a high expression in epithelial cells in endo-/exocrine secretory tissues and mucosa. There is also accordance between localization of mRNA transcripts and detection of proteins, showing that local synthesis likely is responsible for peripheral localization and eventual formation of the CL-LK complexes. The functional implications of the high expression in endo-/exocrine secretory tissue and mucosa is unknown but might be associated with the activity of MASP-3, which has a similar pattern of expression and is known to potentiate the activity of the alternative complement activation pathway.

## Introduction

The innate immune system is a first line of defense and plays major roles in preventing microorganisms in settling and becoming pathogens, and also in the mounting of suitable immune responses against eventual pathogens. It often relies on recognition and binding to pathogen-associated molecular patterns by host pattern recognition molecules (PRMs). Based on an often observed association between microbial evasion of the complement system and pathogenicity (1), the complement system appears to play substantial roles in the innate immune system. The overall antimicrobial functions of the complement system are to tag microorganisms for elimination by phagocytes, to initiate inflammation and to lyse the microorganisms. The complement system is a “self-amplificative“ cascade system, mainly found in the blood, and includes several PRMs; among which some activate a pathway known as the lectin activation pathway, *via* activation of serine proteases known as MBL-associated serine protease (MASP-1, -2, and -3) (2, 3). Mannan-binding lectin (MBL) is probably the most studied PRM of the lectin activation pathway and binds to mannose-rich glycoconjugates on the surface of microorganisms, initiating complement activation. In humans, MBL deficiency may increase susceptibility toward infections in certain situations but not in general (4), most likely contributed by coincidental activation of different complement activation pathways by a given microorganism.

Collectin liver 1 (CL-L1) and collectin kidney 1 (CL-K1) are “MBL-like” proteins that also are found in the circulation in association with MASPs and with specificity toward mannose-rich glycoconjugates and negatively charged molecules (5–10). In the circulation they can be found as heteromeric molecules, referred to as CL-LK, with superior complement abilities *via* MASP-2 in comparison with their respective homomers (11). They are both synthesized in the liver by hepatocytes, in the adrenal glands and in the tubules of the kidney, in addition to other tissues as well (5–7). On the protein level, human CL-K1 has been associated with the same tissues, while human CL-L1 in the original study only was associated with hepatocytes, however, without examination or exclusion of other tissues and cells (6, 7). Among normal healthy populations of different origins, they both constitute average serum concentrations of 250–450 ng/ml, with a clear correlation between levels of CL-L1 and CL-K1, supportive of heteromeric complexes between the two or similar regulation (7, 12–15). The liver and adrenals are due to their endocrine nature and a relative high synthesis of mRNA believed to be the major organs contributing to the CL-L1 and CL-K1 found in the circulation. Partly due to the recently described characterization and association with complement, little is known about their roles *in vivo*. In a recent work using mice deficient of CL-K1, Wakamiya and colleagues showed that CL-K1 protected mice against *Streptococcus pneumonia* infections induced *via* nasal inoculation (16). However, in another work there was no protective effect of CL-K1/CL-LK in a mouse model for infection by *Mycobacterium tuberculosis* (17). CL-K1 has been shown to bind with relative high affinity to the disaccharide Man(α1-2) and to negatively charged molecules, including nucleic acid ligands, and may also play a role in the opsonization of apoptotic cells by recognizing a combination of carbohydrate and nucleic ligands (10, 18).

Recently, it was demonstrated that CL-K1-deficient mice partly were protected against destructive complement-mediated inflammatory responses in post ischemic kidneys and that CL-K1 further promoted development of renal fibrosis in the tubules (19, 20).

CL-K1 and CL-L1 are not regulated significantly by inflammatory stimuli. Their plasma/serum levels do not correlate with increased levels of traditional inflammatory mediators, including CRP and TNF-alpha (8, 9).

The two collectins play apparently also important roles for embryogenesis. Deficiency of CL-K1 or CL-L1 leads in humans to a rare congenital developmental syndrome known as 3MC (alias Malpuech facial clefting syndrome), an effect that the two collectins share with MASP-3. It has been shown that CL-K1 and CL-L1 may act as attractants and guidance cue for neural crest cells, although the precise mechanism for embryonic involvement remains to be elucidated (21, 22).

A functional and complete activation of the complement system involves many complement factors and is in general only associated with the effect in the circulation or at inflamed sites, where blood components gain access. Most tissue expresses certain complement components, e.g., the lung and the intestinal system (23, 24). At such sites the complement system is believed to be partially functional or to mediate activation when inflammation progresses. In the quest of elucidating the function of CL-L1 and CL-K1, we characterized their localization in human tissues. It appears that the previously characterized sites of localization in the circulation, adrenals, liver, and kidney, may have disparaged a compelling localization in especially exocrine/endocrine tissues and mucosa, suggestive of that CL-L1, CL-K1, and CL-LK may play roles in the periphery as well.

## Materials and Methods

### General Reagents and Buffers

Unless otherwise stated, reagents were obtained from Sigma-Aldrich, Denmark. Phosphate-buffered saline: 10 mM Na_2_HPO_4_, 140 mM NaCl, and 2.7 mM KCl pH 7.4. Tris-buffered saline (TBS): 10 mM Tris and 145 mM NaCl. TNT buffer: 0.10 M Tris, 0.15 M NaCl, and 0.05% Tween 20 pH 7.5.

### Generation of MAbs Against CL-L1 and CL-K1

CL-LK was purified from outdated plasma by calcium sensitive immunoaffinity chromatography as previously described (25). Purified CL-LK (50 µg) was used for *s.c*. immunizations of outbred NMRI female mice using Gerbu as adjuvant. Three days before the fusion, mice were boosted (i.p.) with the same amount of CL-LK. The fusion between spleen cells obtained from the CL-LK immunized mice and myeloma cells (Sp2) was performed using polyethylene glycol essentially as described previously in Ref. (26). Positive clones were identified by ELISA using microtiter plates coated with either purified recombinant CL-K1 or CL-L1 expressed in CHO cells as full-length molecules without any tags. Cells from the positive wells were recloned at least thrice by the limiting dilution method. For antibody production and subsequent purification, hybridomas were grown and allowed to express the MAb in Hybridoma-SFM (Invitrogen). Monoclonal antibodies were purified by means of affinity chromatography using a HiTrap Protein G HP column (GE Healthcare) under previously described conditions and elution with 50 mM glycine, pH 2.3 (27). The two antibodies, MAb 11-1 (anti-CL-K1) and MAb 16-13 (CL-L1), which were superior in specificity and IHC sensitivity and applied in the following studies, were both of the isotype IgG1kappa.

### SDS-PAGE and Western Blotting

SDS-PAGE was performed according to the method of Laemmli (1970) using pre-casted NuPAGE 4–12% Bis-Tris gels (Invitrogen) and MES or MOPS SDS running buffer (Invitrogen) (28). Proteins were transferred to the Hybond-P polyvinylidene fluoride membrane (GE Healthcare) (29). The membrane was blocked in 5% non-fat dried milk and 0.1% HSA, and incubated with primary monoclonal antibodies (0.5 µg/ml). Subsequently, the membrane was washed and incubated with HRP-conjugated rabbit anti-mouse antibody diluted (1:20,000) accordingly to the manufacturer’s recommendation (Dako, Denmark) and developed by means of the ECL plus Western blotting detection kit (GE Healthcare). For specificity testing of applied antibodies, 1 µl of serum was applied to the gel per 4 mm well width.

### Surface Plasmon Resonance

Binding characteristics of the monoclonal antibodies, 11-1 and 16-13, was investigated by SPR on a Biacore 3000 instrument (Biacore, Sweden) with immobilized CL-K1, CL-L1, and CL-LK on a CM5 chip. The proteins were immobilized on EDC/NHS-activated flow cells by injecting the collectin (10 µg/ml) in 10 mM acetate pH 5.0 to a surface density ranging from 0.028 to 0.042 pmol/mm^2^. One M of ethanolamine, pH 8.5 was used for capping. For binding experiments antibodies were diluted in running buffer (10 mM HEPES, 150 mM NaCl_2_, 5 mM EDTA, and 0.005% Tween-20 pH 7.4) in a range from 2.08 to 166 nM. Each sample (40 µl) was injected with a flow rate of 5 µl/min. Regeneration was obtained for antibody 11-1 by injection of two cycles of 10 µl: 10 mM glycine, 20 mM EDTA, 500 mM NaCl, and 0.005% Tween-20 pH 4.0. Regeneration was obtained for antibody 16-13 by injection of two cycles of 10 µl: 100 mM glycine, 5 mM EDTA, 500 mM NaCl, and 0.05% Tween-20 pH 3.0. BIAevaluation software 4.0.1 was used for analysis of the data. The apparent dissociation constants were found by fitting the curves to a 1:1 binding model. All experiments were as a minimum conducted as triplicates of duplicates.

### mAbs’ Impact on Ligand Binding and Complement Activation

MaxiSorpTM 96-well plate were coated with DNA (2 µg/ml, cat. no. D2001, Sigma-Aldrich) or mannan (20 µg/ml, cat. no. M3640, Sigma-Aldrich) in 1 M NaCl. Purified native CL-LK (0.25 µg/ml) was pre-incubated in non-adsorbent wells for 1 h at RT in the presence of serum, mannose, or mAbs, diluted twofold from 10%, 200 mM, or 2 µg/ml respectively, and subsequently incubated on ligand-coated plates for 4 h. Bound CL-LK was detected using biotin-labeled anti-CL-K1 mAb (0.5 µg/ml, Hyb 14-29, N-terminal specific) and streptavidin-HRP (0.1 µg/ml). Samples were diluted in TBS (20 mM Tris, 125 mM NaCl, pH 7.4) with 2 mM CaCl2, 0.05% Tween 20, and 0.1% BSA. For assessing CL-LK-mediated complement activation plates were prepared as above. Purified native CL-LK (2 µg/ml) was pre-incubated in non-adsorbent wells with mAbs (0.5 or 4 µg/ml) or mannose (50 or 200 mM) at 37°C for 1 h, and subsequently incubated on ligand-coated plates at 37°C overnight. Wells were washed and incubated with recombinant MASP-2 (0.25 µg/ml for 3 h) followed by incubation with purified C4 (4 µg/ml, CompTech, Tyler, TX, USA, for 1 h) at 37°C. C4b deposition was detected using biotinylated anti-C4 mAb (0.5 µg/ml, HYB 162-02, BioPorto, Gentofte, Denmark). Samples were diluted in VBS (5 mM barbital, 142 mM NaCl, pH 7.4) with 2 mM CaCl_2_, 1 mM MgCl_2_, 0.05% Tween 20, and 0.1% BSA.

### Human Tissue Samples

Human tissue samples were obtained from the tissue bank at the Department of Pathology, Odense University Hospital (Odense, Denmark) and derived from surgically removed specimens fixed 4% phosphate buffered formaldehyde for 12–48 h. Samples were conventionally dehydrated, and subsequently embedded in paraffin before sectioning (4–5 µm) and mounting on slides.

### Immunohistochemistry

Paraffin-embedded, formalin-fixed human tissue sections were deparaffinized and rehydrated through serial wash in xylene and decreasing concentrations of ethanol. Endogen peroxidase activity was blocked by incubation with 1.5% H_2_O_2_ for 10 min. Antigen retrieval was performed by microwave boiling in 10 mM Tris base, 0.5 mM EDTA, and pH 9.0 buffer for 15 min. The tissue sections were washed in TNT buffer and incubated with primary antibodies MAb 16-13 (2 µg/ml), 11-1 (0.5 µg/ml), and mouse MAb anti-chicken IgY (1 µg/ml) for 1 h. The tissue sections were washed in TNT buffer and incubated with EnVision + System HRP-labeled polymer (Dako) for 30 min. After wash in TNT buffer, the tissue sections were incubated with DAB+ (Dako) for 10 min followed by staining with hematoxylin. The final immunohistochemical analysis was carried out using “multi block” sections comprising the following normal tissues: the cerebellum, esophagus, fetal and adult liver, gall bladder, kidney, large intestine, lung, skeletal muscle, pancreas, parotid gland, placenta, prostate, pylorus, spleen, tonsils, thymus, thyroid gland, rectum, small intestine, testis, and urinary bladder. The adrenal gland was derived from a patient diagnosed with pheochromocytoma.

### Image Acquisition

Histology slides were scanned at 20× (controls) or 40× magnification using a NanoZoomer-XR (Hamamatsu Photonics, Japan). Image sections were acquired using NDP.view2 software (NanoZoomer Digital Pathology; Hamamatsu Photonics) and final JPG images were all uniformly adjusted for color saturation (+25) and light (−1) in Adobe Photoshop.

## Results

### Antibody Specificity, Affinities, and Impact on Complement Activation

**Figure 1:**
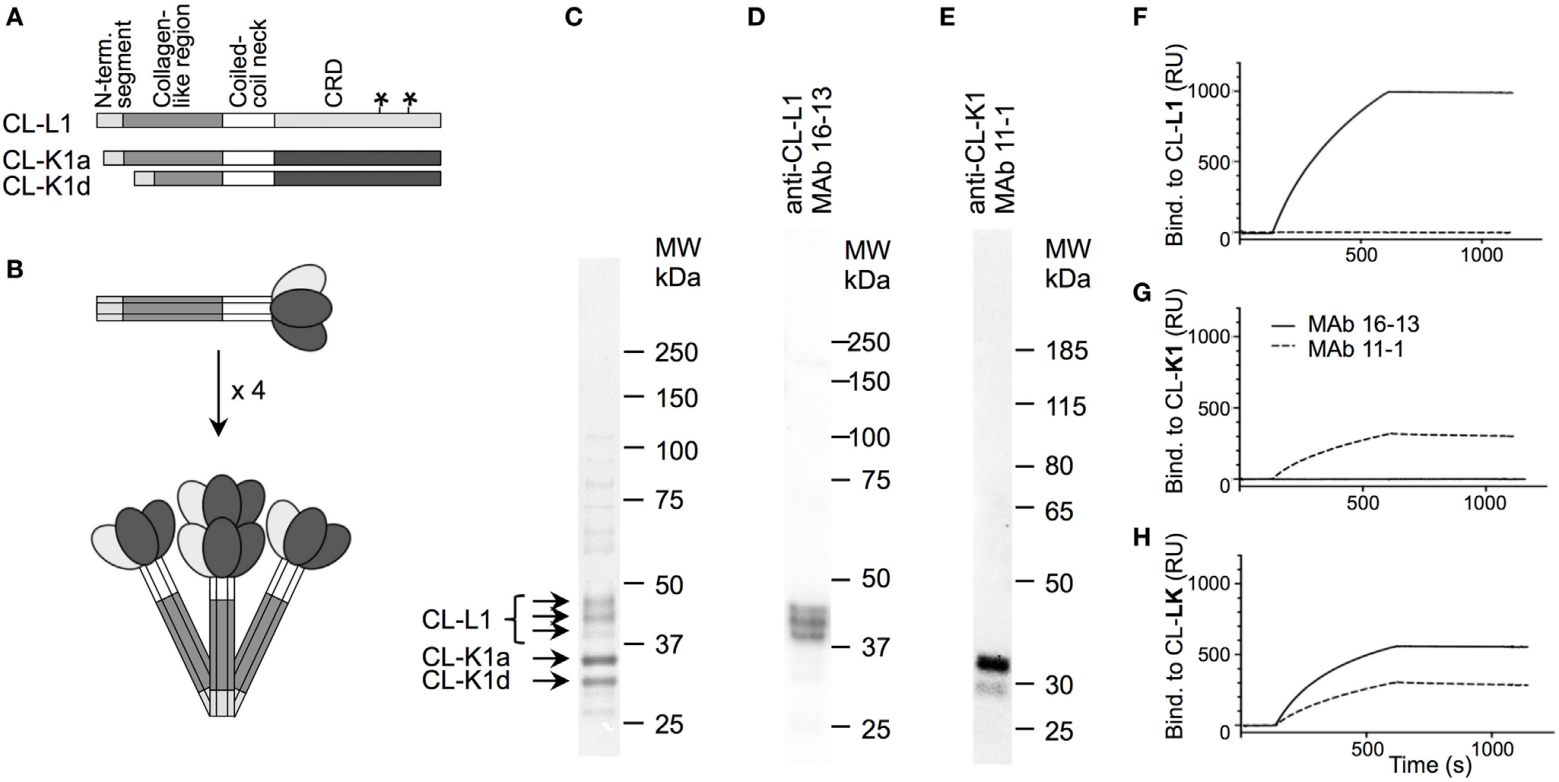
Structure of CL-LK and antibody specificity. **(A)** Schematic illustration of the domain organization of CL-L1 and CL-K1 polypeptide chains. The symbol “*”on the CL-L1 polypeptide chain represent two N-linked glycosylation sites in the carbohydrate recognition domain. CL-K1 is found in the circulation in the form of two isoforms: CL-K1a represents full-length and CL-K1d represents an alternative spliced form devoid of a part of the collagen-like region. **(B)** Subunit of CL-LK and oligomeric structures. A total of three polypeptide chains of CL-K1 and CL-L1 join to form a heteromeric subunit, which may further oligomerize into structures ranging from dimers to hexamers of subunits, here illustrated by a tetramer. **(C)** Analysis of purified CL-LK by SDS-PAGE and visualization by silver staining. The three bands of CL-L1 represent non-, partially, and fully glycosylated forms of CL-L1. **(D,E)** Specificity of monoclonal antibodies by Western blotting of serum under. reducing conditions and visualization by ECL. **(F–H)** SPR analyses of monoclonal antibodies with immobilized CL-K1, CL-L1, and CL-LK as antigen, respectively. MAbs 16-13 (**--**) and 11-1 (**---**) were analyzed for binding to immobilized purified CL-L1 **(F)**, CL-K1 **(G)**, or CL-KL **(H)** MAb using concentrations ranging from 0.1 to 10 µg/ml.

**Figure 2:**
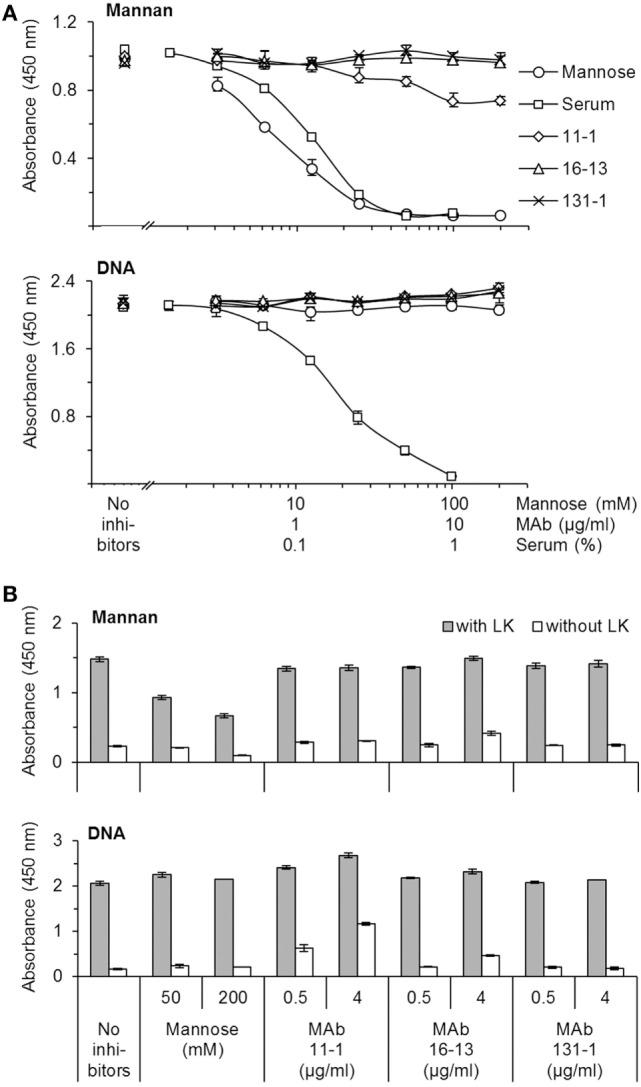
Characterization of mAbs’ impact on the ligand-binding and complement activity mediated by CL-LK. **(A)** Impact on ligand-binding. CL-LK was incubated on ligand-coated plates (mannan or DNA) in the presence or absence of the mAbs used for IHC (mAb 11-1 and mAb 16-13), mannose, or serum (known to interfere with the binding of CL-LK to ligands). mAb 131-1 (anti-MBL) was included as control. Bound CL-LK was detected with biotinylated mAb-anti-CL-K1 (compatible with the applied mAbs) and HRP-streptavidin. **(B)** Impact on CL-LK-mediated complement activation. Prior to incubation with MASP-2 and C4, plates were prepared with CL-LK and coated ligands (mannan or DNA) as above. Deposition of C4b was detected with biotin-labeled anti-C4b mAb and HRP-streptavidin. The results shown are representative of three independent experiments. Error bars refer to max and min of triplicate measurements. None of the tested mAbs interfered with ligand binding or complement activation. CL-LK binding to mannan and DNA occurs *via* two separate binding site, and the latter is not inhibited by mannose, whereas uncharacterized blood components inhibit both types of interaction (10, 11).

The reactivity of the applied MAbs was demonstrated by Western blotting using serum as source of antigens (Figure 1). This analysis showed that the applied MAbs 16-13 (anti-CL-L1) and 11-1 (anti-K1) only reacted with protein bands correlating with the molecular weight of CL-L1 and CL-K1, respectively (Figure 1) (7). There was no cross-reactivity of the two antibodies, and both MAbs recognized all isoforms, ensuring detection of all forms of CL-K1 and CL-L1 in the tissue sections. To further validate the specificity and reactivity, the two monoclonal antibodies were analyzed by SPR using immobilized purified collectins and antibodies in fluid phase. MAb16-13 bound to CL-L1 (*K_D_* = 0.16 ± 0.007 nM, means ± SD) and to CL-LK (*K_D_* = 0.14 ± 0.003 nM) but not to CL-K1. MAb 11-1 bound to CL-K1 (K1 *K_D_* = 5.4 ± 1.8 nM) and to CL-LK (*K_D_* = 4.6 ± 1.9 nM) but not to CL-L1. Again, cross-reactivity was undetectable and binding affinities/avidities were of satisfactory strengths. In the characterization of the two applied MAbs, we found that they neither interfered with the binding activity of CL-LK to suitable ligands nor did they modulate or inhibit the CL-LK-mediated complement activation *via* MASP-2 (Figure 2).

### Immunohistochemical Localization of CL-L1 and CL-K1

In the majority of the tested tissues we observed identical localization of CL-K1 and CL-L1, both in terms of tissue and cell types. Unless the difference in immunoreactivity between the two was striking, the co-localization is not commented further, neither is the absence of staining of the tissues incubated with isotype matched control antibody. Frequently, the immunoreactivity of the CL-K1 MAb (11-1) was stronger than that of the CL-L1 MAb (16-13). This may not necessarily reflect an increase in CL-K1 quantity in comparison with CL-L1 but may originate from the nature of the antibodies (also discussed further below).

**Figure 3:**
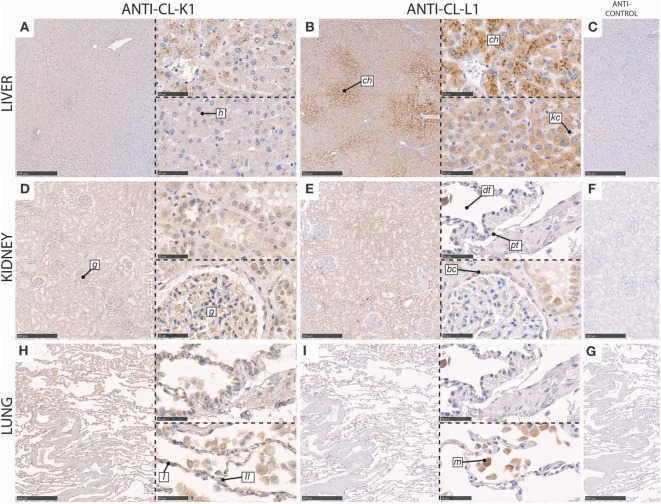
Immunohistochemical localization of CL-K1 and CL-L1 in formalin fixed and paraffin embedded sections of the liver **(A,B)**, kidney **(D,E)**, and lung **(H,I)**. Italic letters within images refer to: for the **liver** *h*: hepatocytes, *ch*: centrilobular hepatocytes, and *kc*: Kupffer cells; **kidney** *g*: glomerulus, *dt*: distal tubules, *pt*: proximal tubules, and *bc*: Bowman’s capsule; **lung** *I*: type I pneumocytes, *II*: type II pneumocytes, and *m*: alveolar macrophages. Scale bars in large sections and in isotype control sections **(C,F,G)** correspond to 500 µm and in small sections to 50 µm.

In the liver, immunoreactivity for CL-K1 and CL-L1 was associated with hepatocytes with absent staining of Kupffer cells. Staining intensities of CL-L1 was pronounced in the centrilobular hepatocytes (Figure 3).

In the kidney, immunoreactivity for CL-K1 and CL-L1 was especially associated with the tubular system, with the most pronounced staining of the distal tubules (Figure 3), in comparison with proximal tubules. CL-L1 immunoreactivity was for some distal tubules distinctly associated with the brush border. Immunoreactivity for both collectins was also associated with the epithelial cells lining the Bowman’s capsules, whereas immunoreactivity in the glomerulus itself mainly was associated with CL-K1 and only minimally with CL-L1. CL-K1 immunoreactivity in the glomerulus was associated morphologically appeared to include both podocytes and mesangial cells.

In the lung, CL-K1 immunoreactivity was associated with alveolar macrophages, type I and II pneumocytes (Figure 3). CL-L1 immunoreactivity appeared only to be associated with alveolar macrophages.

**Figure 4:**
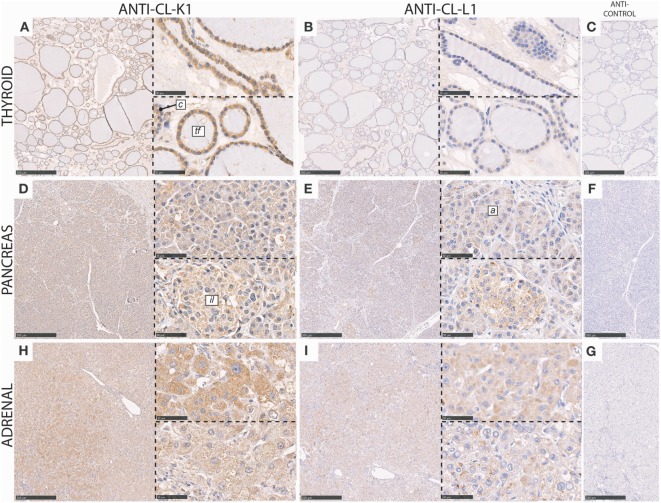
Immunohistochemical localization of CL-K1 and CL-L1 in formalin fixed and paraffin embedded sections of the thyroid gland **(A,B)**, pancreas **(D,E)**, and adrenal gland from a patient diagnosed with pheochromocytoma, **(H,I)**. Italic letters within images refer to: for the **thyroid gland** *tf*, thyroid follicles and *c*, parafollicular C cells; **pancreas** *il*: islets of Langerhans and *a*: acinar epithelial cells. Scale bars in large sections and in isotype control sections **(C,F,G)** correspond to 500 µm and in small sections to 50 µm.

In the thyroid gland, cuboidal epithelial cells lining the base membrane of thyroid follicles and parafollicular cells (C-cells) were associated with immunoreactivity for both CL-K1 and CL-L1 (Figure 4). Most pronounced staining was observed for CL-K1.

In the pancreas CL-K1 and CL-L1 immunoreactivity was associated with the islets of Langerhans and the pancreatic epithelial acinar cells and ducts (Figure 4). Within the islets, the vast majority of cells stained positive, indicating for sure that insulin-producing cells (beta cells) were associated with immunoreactivity and also most likely glucagon-producing cells (alpha cells) as well.

In the adrenal tissue section (Figure 4), derived from a patient diagnosed with pheochromocytoma, the histology was slightly unclear. However, as immunoreactivity for both CL-K1 and CL-L1 was associated with nearly all cells, it was deducted that the majority of adrenal cells, including both medullary and cortical cells, are associated with the two collectins, similar with previous findings for the localization of CL-K1 (7).

**Figure 5:**
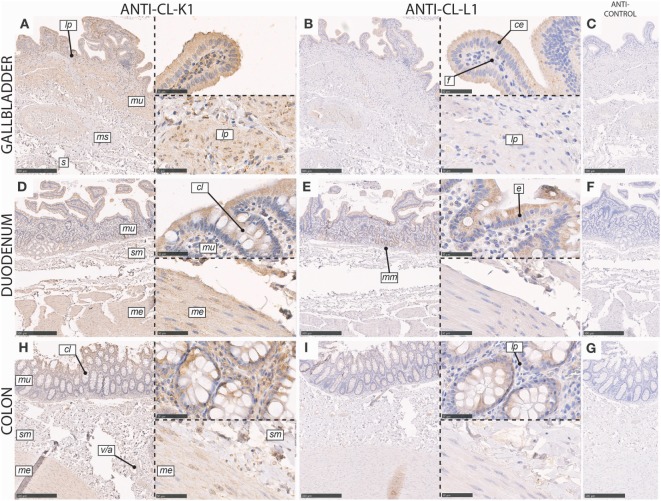
Immunohistochemical localization of CL-K1 and CL-L1 in formalin-fixed and paraffin-embedded sections of the gallbladder **(A,B)**, duodenum **(D,E)**, and colon **(H,I)**. Italic letters within images refers to: for the **gallbladder** *s*: serosa layer, *ms*: muscularis layer, *m*: mucosa layer, *lp*: lamina propria, *ce*: columnar epithelial cells, and *f*: fibroblasts; **duodenum** and **colon** *me*: muscularis externica layers, *sm*: submucosa, *mu*: mucosa, *cl*: crypts of Lieberkuhn (tubular glands), *mm*: muscularis mucosa, and *v/a*: vein/artery. Scale bars in large sections and in isotype control sections **(C,F,G)** correspond to 500 µm and in small sections to 50 µm.

In the gall bladder, immunoreactivity for both CL-K1 and CL-L1 was associated with columnar epithelial cells of the mucosal folds, with increasing intensity toward the luminal side of the folds (Figure 5). Various cell types in the lamina propria stained weakly positive. We observed only scattered staining of cells in the muscularis and serosa layers.

In the duodenum, immunoreactivity for CL-K1 and CL-L1 was associated with epithelial cells in both the mucosa and submucosa. In the mucosal luminal membrane of the villi, especially columnar cells (enterocytes) stained positive (Figure 5). Further and intense immunoreactivity of the mucosa was associated with the crypts of Lieberkuhn, whereas the muscularis externa was only weakly positive for staining. In the submucosa, immunoreactivity was associated with cells of the Brunner’s glands.

In the colon, CL-K1 and CL-L1 immunoreactivity was dominantly associated with mucosa and especially with columnar epithelial cells in the crypts of Lieberkuhn (tubular glands) (Figure 5). In the lamina propria, the staining was scattered and associated with various cells types. Within the layers of the muscularis externa and submucosa, staining was also associated with endothelial cells, best illustrated for the localization of CL-K1.

**Figure 6:**
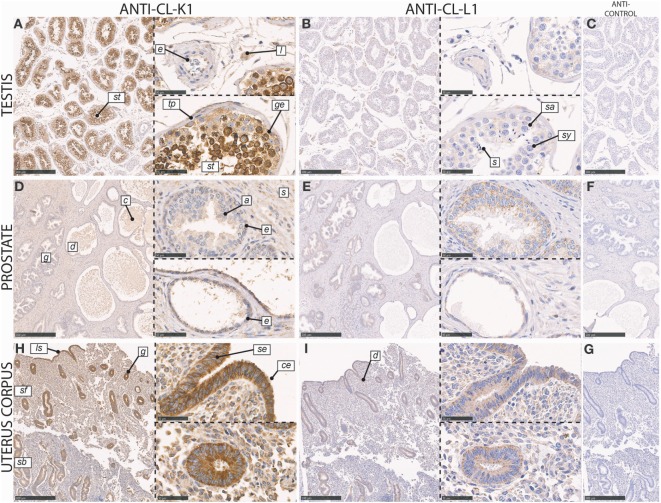
Immunohistochemical localization of CL-K1 and CL-L1 in formalin-fixed and paraffin-embedded sections of the testis **(A,B)**, prostate **(D,E)**, and uterus corpus **(H,I)**. Italic letters within images refers to: for the **testis** st: seminiferous tubules, *e*: endothelial cells, *l*: Leydig cell, *tp*: tunicapropria, *ge*: germinal epithelial cells, *s*: spermatogonia, *sy*: spermatocytes, and *s*: spermatids; **prostate** *g*: gland, *d*: duct, *c*: “concretized” material: *a*: acininar cells/acinus, *s*: stroma, and *e*: basal epithelial cells; **uterus corpus** *sb*: stratum basale, *sf*: stratum functionalis, *ls*: luminal surface, *g*: gland, *d*: duct, *se*: stratified columnar epithelial cells, and *ce*: ciliated epithelial cells. Scale bars in large sections and in isotype control sections **(C,F,G)** correspond to 500 µm and in small sections to 50 µm.

In the testis, CL-K1 immunoreactivity was associated with germinal epithelial cells lining the tunica propria of the seminiferous tubules, spermatogonia (type A and B), and spermatocytes (primary and secondary) (Figure 6). These cells were embedded in the less immunoreactive Sertoli cells. In the interstitium between seminiferous tubules, Leydig cells and endothelial cells of capillaries stained weakly positive. CL-L1 immunoreactivity was weak in comparison with that of CL-K1, but the pattern of the two collectins followed each other.

In the prostate, CL-K1 and CL-L1 immunoreactivity was associated with epithelial cells of the prostatic glands, with staining of both acini and ducts (Figure 6). The staining was associated with both columnar pseudostratified and involuted luminal epithelial cells; however, with most intense staining of the basal epithelial cells. In the stroma, scattered staining was observed in various cells types, including staining of endothelial cells. In the ducts, secretory vesicles and concretized material stained weakly positive, especially for CL-K1.

In the corpus uterus, CL-K1 and CL-L1 immunoreactivity was localized to the epithelial cells in the endometrial glands, glandular ducts, and at luminal surface, with comparable staining of glandular structures in both the stratum functionalis (compactum and spongiosum) and stratum basale (Figure 6). Both stratified columnar and ciliated cells in the glands stained intensely. In the stroma, the immunoreactivity was moderate but associated with the majority of cells, with a pronounced staining of endothelial cells of the capillaries.

**Figure 7:**
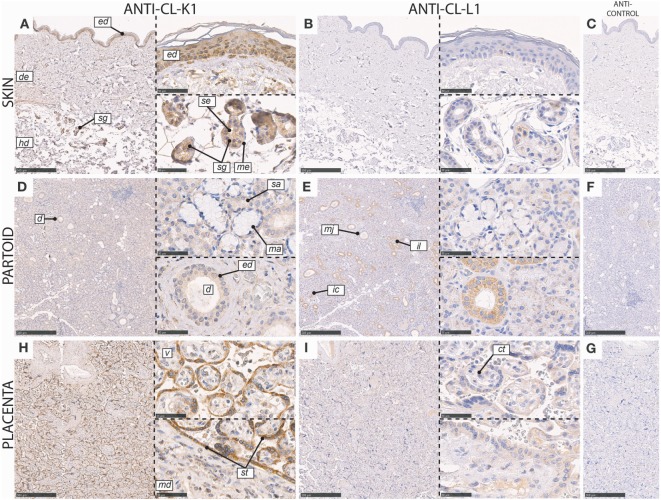
Immunohistochemical localization of CL-K1 and CL-L1 in formalin fixed and paraffin embedded sections of the skin **(A,B)**, parotid gland **(D,E)**, and placenta **(H,I)**. Italic letters within images refers to: for the **skin** *ed*: epidermis, *de*: dermis, *hd*: hypodermis, *sg*: sweat glands, *me*: myoepithelial cells, and *se*: stratified cuboidal epithelia cells; **partoid salivary gland** *d*: duct, *ed*: epithelial ductal cells, *sa*: serous acini, *ma*: mucous acini, *mj*: major duct, *il*: intralobulated duct, and *ic*: intercalated duct; **placenta** *v*: villus, st: syncytiotrophoblast, *ct*: cytotrophoblast, and *md*: mesoderm. Scale bars in large sections and in isotype control sections **(C,F,G)** correspond to 500 µm and in small sections to 50 µm.

In the skin, CL-K1 and CL-L1 immunoreactivity was associated with the sweat glands and ducts (Figure 7). CL-K1 staining was further associated with the basal layer of the epidermis. In the sweat glands and ducts, especially epithelial cells, stained positive, while myoepithelial cells only stained weakly positive. Within the inner duct, staining was associated with the luminal part and eventual content in the duct. Staining of sporadically distributed and non-identifiable cells in the dermis was also observed.

In the partoid salivary glands, immunoreactivity for CL-L1 and CL-K1 was associated with both epithelial glandular (acini) and epithelial ductal cells (Figure 7). Immunoreactivity was localized dominantly to the epithelial cells constituting the salivary ducts and less with the secretory acini. However, the majority of serous-producing epithelial cells were associated with immunoreactivity. Basal epithelial cells of mucin producing stained weakly positive. All three kinds of ducts: intercalated (minor), intralobulated (striated), and major showed equal and dominant immunoreactivity. The immunoreactivity of CL-L1 in the salivary serous glands was superior to that of CL-K1.

In the full-term (mature) placenta, CL-K1 and CL-L1 immunoreactivity was mainly associated with the syncytiotrophoblast layer, at the border of maternal and fetal circulation, and weakly with the underlying cytotrophoblasts associated with a villus (Figure 7).

Various levels of CL-K1 and CL-L1 immunoreactivity were also found to be associated with the following tissues: the thymus, spleen, tonsil (Figure S1 in Supplementary Material), esophagus, stomach (Figure S2 in Supplementary Material), cervix uterus, portio uterus, abortus (Figure S3 in Supplementary Material), skeletal muscle, cerebellum, and urinary bladder (Figure S4 in Supplementary Material). A description of their detailed localizations is provided in the associated figure legends.

### CL-K1 and CL-L1 mRNA Abundancies

To compare protein localization by IHC with site of synthesis for the two collectins, data were retrieved from the three major RNA expression databases HPA, GTEx, and FANTOM5 RNA. For comparison, expression levels were normalized and graduated into four categories based on a logarithmic division (Tables S1 and S2 in Supplementary Material). Levels of immunoreactivity were visually validated by three independent persons and categorized similarly (Table 1).

**Table 1:** Levels of RNA expression: The symbols “+++,” “++,” “+,” and “– ”denote high, medium, low, and absent/extremely low expression, respectively, based on the criteria established in Figures S1 and S2 in Supplementary Material.

	*CL-K1* IHC/RNA^a^	*CL-L1* IHC/RNA^a^	*MASP-1* IHC/RNA^b^	*MASP-3* IHC/RNA^b^	*MAp44* IHC^c^/RNA^d^	*MASP-2* IHC^e^/RNA^f^	*MAp19* IHC^e^/RNA^e^	*MBL* IHC^g^/RNA^a^
Abortus	++/	+/						
Adrenal	+++/+++	++/+						−/−
Cerebellum	++/+	+/−					+/	−/−
Cervix uterus	++/+	+/−	/+	/+++	/++	/−	/−	−/−
Colon	++/+	+/−	/−	/+++	/+	+/−	/−	−/−
Corpus uterus	+++/	+/						
Esophagus	++/+	−/−	/++	/+	/−	/−	/−	−/−
Fetal liver	+/	++/						
Fetal muscle	++/	++/						
Gallbladder	+++/+++	++/+						+/−
Heart	/++	/+	/++	/+++	+++/+++	/−	/−	+/−
Kidney	++/++	++/+	/++	/++	/−	++/−	++/++	+/−
Liver	++/+++	+++/+++	/+++	/+++	+++/++	+++/+++	+++/+++	+++/+++
Lung	++/+	+/+	/++	/+++	/++	+/+	++/−	−/−
Muscle	+/+	−/−	/−	/+++	++/+++	/−	/−	−/−
Pancreas	++/++	+/-	/−	/+++	/−	+/−		−/−
Placenta	+++/++	+/+++	/++	/+++	/−	/−	/−	−/−
Portio uterus	+/	−/						
Prostate	++/+	+/+	/−	/+++	/−	/−	+/−	−/−
Salivary gland	+/+	++/−						−/−
Skin	++/+	+/−						−/−
Small intestine	++/++	+/+	/++	/+++	/−	/++	/−	−/−
Spleen	++/++	+/−	/−	/++	/−	/−	/−	−/−
Stomach	++/+	+/−					+/	+/−
Testis	+++/++	+/−	/−	/++	/−	/++	/−	−/−
Thymus	+/	−/	/−	/++	/−	/−	/−	
Thyroid	+++/++	+/+	/−	/−	/−	/−	+/+++	−/−
Tonsil	+/+	−/−						−/−
Urinary bladder	++/++	−/+	/−	/++	/−	/−	/−	−/−

*IHC immunoreactivity levels were judged arbitrarily into the same categories by three independent validations (persons). Missing symbols reflect that the tissue has not been analyzed. MASP-1, MASP-3, and Map44 derive all from the MASP1 gene. MASP-2 and MAp19 derive both from the MASP2 gene*.

*^a^Relative and normalized data from HPA, GTEx, and FANTOM5 RNA expression databases (Tables S1–S3 in Supplementary Material)*.

*^b^Relative and normalized data from Seyfarth et al. (30) and Degn et al. (31) (Tables S4 and S5 in Supplementary Material)*.

*^c^Skjoedt et al. (32)*.

*^d^Relative and normalized data from Degn et al. (31) and Skjoedt et al. (32) (Table S6 in Supplementary Material)*.

*^e^Degn et al. (33) (Table S7 in Supplementary Material)*.

*^f^Relative and normalized data from Seyfarth et al. (30) and Degn et al. (33) (Table S8 in Supplementary Material)*.

*^g^The Human Protein Atlas Project (antibody CAB016782)*.

mRNA transcripts encoding CL-K1 was detectable in all tested tissues and the major sites of synthesis, grouped in the “high” category, comprised the adrenals, gallbladder, and liver. In all the tested tissues, there was only minimal variance, in terms of a single category shift, i.e., “high” to “medium,” between CL-K1 mRNA levels and immunoreactivity, therefore we considered there to be an excellent accordance between site of CL-K1 synthesis and protein localization. mRNA transcript encoding CL-L1 was not detected in as many tissues as CL-K1. Some tissues were categorized with “extremely low/absent” number of CL-L1 transcripts. However, by IHC quite a lot of these tissues were found to be associated with immunoreactivity, albeit in a “low” degree. The major site of CL-L1 mRNA synthesis was the liver and placenta and in these tissues the protein was also readily detected. Similar to the observations for CL-K1 and using the same criteria, there appeared in general to be accordance between site of CL-L1 synthesis and protein localization (discussed further below).

To associate the protein localization with an eventual local functionality of the two collectins, mediated *via* the presence of MASPs, localization of MASPs (and MAps), and synthesis of their respective mRNAs were evaluated by the same approach. As the major RNA expression databases currently do not take alternatively splicing of the MASP genes into consideration, data were retrieved and gathered from the previous work by Thiel and colleagues and Garred and colleagues (30–33). MASP-3 expression appeared to both overlap and being as widely distributed as the two collectins, whereas the other MASPs and MAp had a restricted pattern of tissue localization, with the liver being a tissue of major synthesis and/or detection: an observation, which also applied for MBL.

## Discussion

The present work describes the localization of CL-L1 and CL-K1 in human tissues as determined by immunohistochemistry and summarizes further their mRNA tissue profiles derived from transcriptome databases. Both CL-L1 and CL-K1 were demonstrated in epithelial cells in a variety of tissue throughout the human body.

Of all the tested MAbs, MAbs 16-13 (anti-CL-L1) and 11-1 (anti-CL-K1) had the best sensitivity and specificity. The two MAbs demonstrated excellent immunoreactivity in the three tissues, the liver, kidney, and adrenals, wherein human CL-K1 and CL-L1 localization previously have been demonstrated (6, 7). The major positive cell types comprised, hepatocytes, renal epithelial cells of tubules, and medullary and cortical cells of the adrenals. In addition to the adrenals, tissues from other endocrine glands, i.e., pancreas, demonstrated a similar convincing excellent immunoreactivity for both collectins, derived from cells in the islets of Langerhans and epithelial cells of the ducts. Exocrine tissues of the digestive system comprising the gallbladder, duodenum, colon, and also partly the stomach and esophagus were also associated with epithelial and mucosal immunoreactivity for generally both collectins. Other exocrine tissues, comprising sex-specific organs, such as the testis, prostate, and uterus, had also excellent to moderate immunoreactivity for both collectins. Among all the analyzed tissues, the testis and uterus appeared to be the two tissues with the relative highest CL-K1 immunoreactivity. Again, epithelial cells and mucosa in the uterus were the major source of immunoreactivity, whereas immunoreactivity in testis was associated with germinal epithelial cells of the tubules. Among the tissues analyzed, the highest CL-L1 immunoreactivity was observed in the liver, followed by the kidney and parotid gland, wherein immunoreactivity was also associated with epithelial cells. Our observation of CL-K1 synthesis in various tissues falls in line with previous work by Wakamiya and colleagues, who by immunofluorescence techniques demonstrated partly overlapping localization of CL-K1 in murine tissues, using a polyclonal anti-mouse-CL-K1 antibody (34). In general, we observed a stronger staining of CL-K1 than of CL-L1. This may reflect that CL-K1 is more abundant than CL-L1, although their levels in the circulation are approximately the same (12), or it may simply be a matter of affinities of the applied MAbs in combination with availability of antigen epitopes on the fixed and embedded tissue sections.

Retrieval and normalization of mRNA levels from three transcriptome databases demonstrated accordance between site of synthesis and protein localization. The only tissue, wherein there appeared to be a notable difference, was for CL-L1 in the salivary gland. By IHC CL-L1 localization was judged to be medium, whereas CL-L1 mRNA appeared to be absent. Other CL-L1-specific antibodies showed varying staining of particular the salivary gland (data not shown), making us hypothesize that the observed disagreement could reflect some sort of uncharacterized alternative splicing of CL-L1 in this tissue.

Throughout the IHC staining it was evident that within the majority of the tissues, the localization pattern of two collectins was identical; meaning that exactly the same cells in a given tissue demonstrated immunoreactivity for both collectins. This is best exemplified when two neighbor sections were mounted and processed, as illustrated, e.g., with the corpus uterus (Figure 6). Thus, in the majority of tissues there is opportunity for the making of CL-LK heteromeric complexes, and as previously described, this structure also appears to be the most thermodynamic stabile conformation (11). In some of the tissues, comprising the thyroid gland, skeletal muscle, skin, urinary bladder, and partly the testis and esophagus, CL-K1 appeared to be present in large excess in comparison with CL-L1, as judged by immunoreactivity; it is likely that CL-K1 homomers will be the dominating form in these tissues. The precise distribution of homomers vs. heteromers in different tissues should not be judged by immunoreactivity and remains thus to be characterized in detail. Although the heteromers are eminent in their association with MASP-2 and C4b deposition, in comparison with the homomers, it is worth emphasizing that both types of homomers interact well with MASP-1/-3, and may mediate downstream complement activation *via* those alone (8, 11).

To further illustrate the presence of heteromers in different tissues we tried to establish a proximity ligation assay using antibodies usable on formalin fixed sections but without convincing results. By using purified and fixed CL-LK it appeared that even the best combination of antibodies partly shadowed for each other in proximity ligation assays and were only capable of detecting the very high oligomers of CL-LK, with a sensitivity of only 0.2 µg of purified CL-LK per ml immobilized onto polylysine-treated object glasses (data not shown).

The overlapping localization of the two collectins in the same cells justifies, with a few exceptions, possible assembly and presence of the heteromeric CL-LK in most tissues. Based on a combination of previous observations and unpublished results by our laboratory, it appears that the oligomeric state of CL-LK depends on the relative content of CL-L1 and the ratio of the CL-K1a/d isotypes (11). As all of our anti-CL-K1 MAbs recognize the two isotypes equally well, the immunohistochemical results does not *per se* allow us to deduct any final conclusions on the variability of oligomers in different tissues. However, tissues with a relative large expression of CL-L1 could potentially favor assembly of CL-LK into large oligomers, ranging from 2 to 6 subunits.

We have previously demonstrated that the binding activity of CL-K1 and CL-LK, and hence also their complement activating ability, in serum/plasma is inhibited by unknown factors (11). This has made it difficult to comprehend the role of the two collectins as *bona fida* activators of complement. In the light of the widespread presence of CL-K1 and CL-L1 in various tissues, it is possible that binding activity in the periphery, in the absence of inhibitory blood components, may be more efficient.

As the hitherto described biological functions of CL-K1 and CL-L1, in terms of complement activation or involvement in embryogenesis, appear to rely on MASPs it is relevant to investigate co-localization with MASPs in the periphery. However, there is a lack of suitable antibodies specific for the three products of the MASP-1 gene, MASP-1/-3, and MAp44, but the summarized mRNA profile presented in Table 1 shows that MASP-3 synthesis, in contrast with all other MASPs and MAps, appears to overlap greatly with the localization of CL-K1 and CL-L1. Thus, it is likely that the role of CL-K1 and CL-L1 in the periphery is mainly mediated *via* MASP-3, which was recently shown to activate profactor D to factor D, and thereby potentiate the alternative pathway and amplification loop (35–37). Although (pro) factor D mainly is synthesized in adipose tissue (hence the alias “adipsin”) various tissues synthesize minor amounts of profactor D, which could be a target for MASP-3 in complex with CL-K1/-L1/-LK, and thereby potentiate the complement amplification loop in the periphery, upon encounter and binding of collectins to suitable (microbial) ligands. Alternatively, the two collectins may in the periphery, and in parallel with MBL and C1q, exert some of their functions by interacting with the metalloproteases bone morphogenic protein 1 and tolloid-like proteases, involved in extracellular matrix assembly and growth factor signaling (38). Interactions between CL-K1, -L1, or -LK with these metalloproteases remain to be investigated.

In the light of our (co-)localization of CL-K1 and CL-L1 to peripheral tissues it appears that the previously focus on their roles in the circulation, liver, and kidney, may have disparaged a compelling localization in especially exocrine/endocrine tissues and mucosa, suggestive of that CL-L1, CL-K1, and CL-LK may play roles on epithelial surfaces in general and in tissue characterized by a high degree of exocytosis. The localization of CL-K1 and CL-L1 reminds also in many ways of the localization of the collectin surfactant protein D (39).

## Author Contributions

SH and MH designed the study and carried out: antibody development, characterization, immunohistochemistry, data analysis, computational bioinformatics, and wrote the paper, on which all authors commented. JA and KB carried out antibody development, characterization, and immunohistochemistry. EH, ON, and HS participated in designing and performing the immunohistochemistry and analyzing data. KS carried out development of antibodies. AS and JG carried out SPR analysis and analyzed data.

## Conflict of Interest Statement

The authors declare that the research was conducted in the absence of any commercial or financial relationships that could be construed as a potential conflict of interest.
