# Supplementary material for: CL-L1 and CL-K1 Exhibit Widespread Tissue Distribution With High and Co-Localized Expression in Secretory Epithelia and Mucosa
Source: Front Immunol. 2018 Jul 31;9:1757. doi: 10.3389/fimmu.2018.01757 (PMC6079254; doi:10.3389/fimmu.2018.01757)
Supplement: Supplementary file 1 [file Data_Sheet_1.PDF]

SUPPLEMENTAL FIGURES AND TABLES

CL-L1 and CL-K1 exhibit widespread tissue distribution with high and co-localized expression in secretory epithelia and mucosa

Soren W. K. Hansen[1,] Josephine B. Aagaard[1], Karen B. Bjerrum[1], Eva K. Hejbøl[2], Ole Nielsen[2], Henrik D. Schrøder[2], Karsten Skjoedt[1], Anna L. Sørensen[1], Jonas H. Graversen[1] and Maiken L. Henriksen[1]

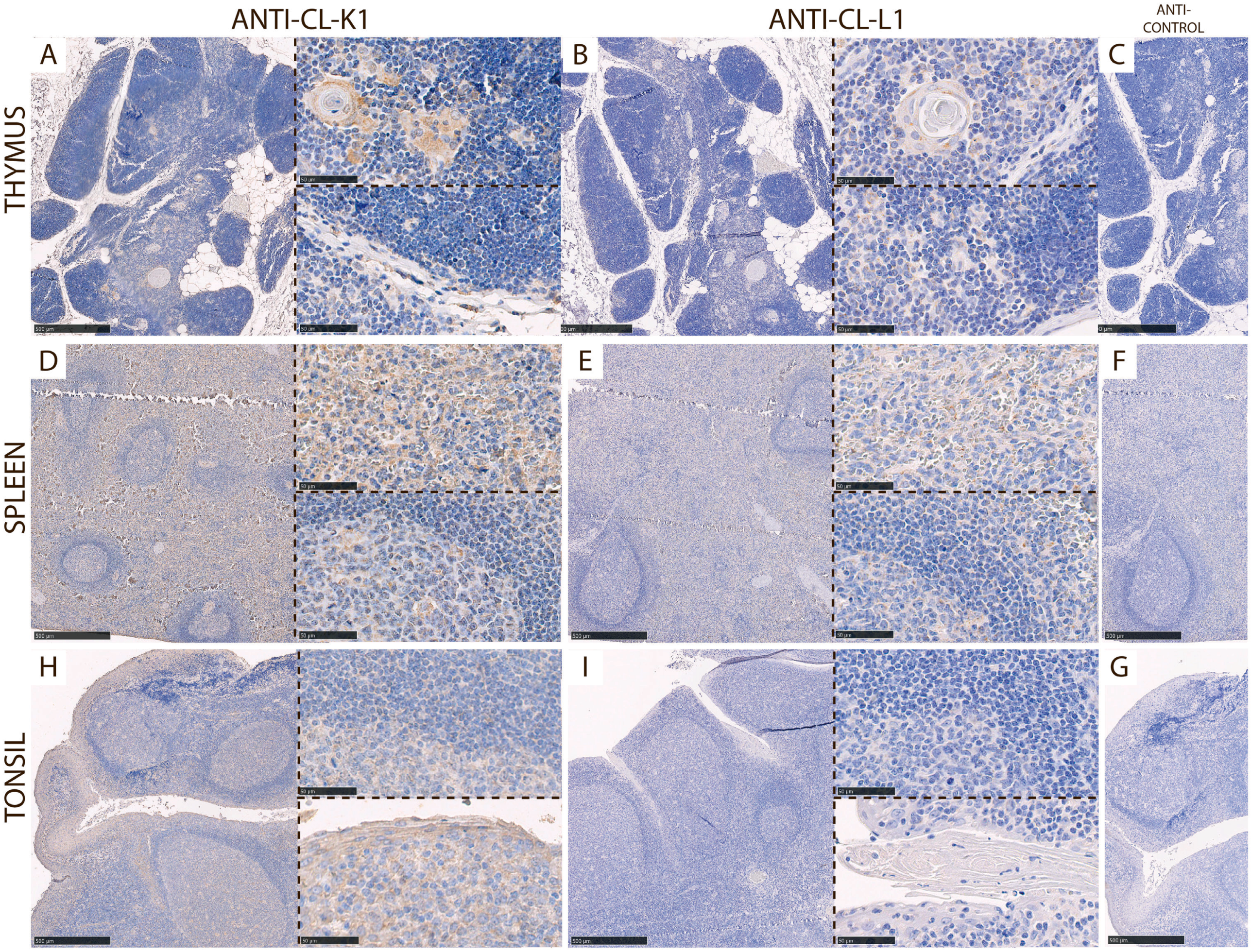

Suppl. figure 1. Immunohistochemical localization of CL-K1 and CL-L1 in formalin fixed and paraffin embedded sections of the thymus (A, B), spleen (D, E) and tonsil (H and I). Scale bars in large sections and in isotype control sections (C, F, G) correspond to 500 µm and in small sections. In **the thymus**, Cl-K1 and CL-L1 immunoreactivity was associated with both the medulla and cortex. In the medulla staining of epithelial reticular cells was dominant and especially in the periphery and surroundings of the Hassall's corpuscles. In the cortex, staining was mainly associated with the outer cortex, with cells, likely macrophages or reticular epithelial cells, close to the corticomedullary junctions. In **the spleen** CL-K1 and CL-L1 immunoreactivity was observed in the white pulp and mainly with lymphoid follicles. In the follicles, germinal centers stained intensely with the surrounding lymphocytes being negative but the marginal zone being positive. In the red pulp, staining was also significant but scattered in comparison with the staining of the white pulp. In **the tonsils**, CL-K1 and CL-L1 immunoreactivity was associated with the germinal centers of the lymphoid nodules and with the crypts. In the germinal centers the majority of lymphoid cells stained positive for especially CL-K1, in comparison with CL-L1. At the crypts, the stratified squamous epithelial cells stained positive for CL-K1 but only weakly for CL-L1.

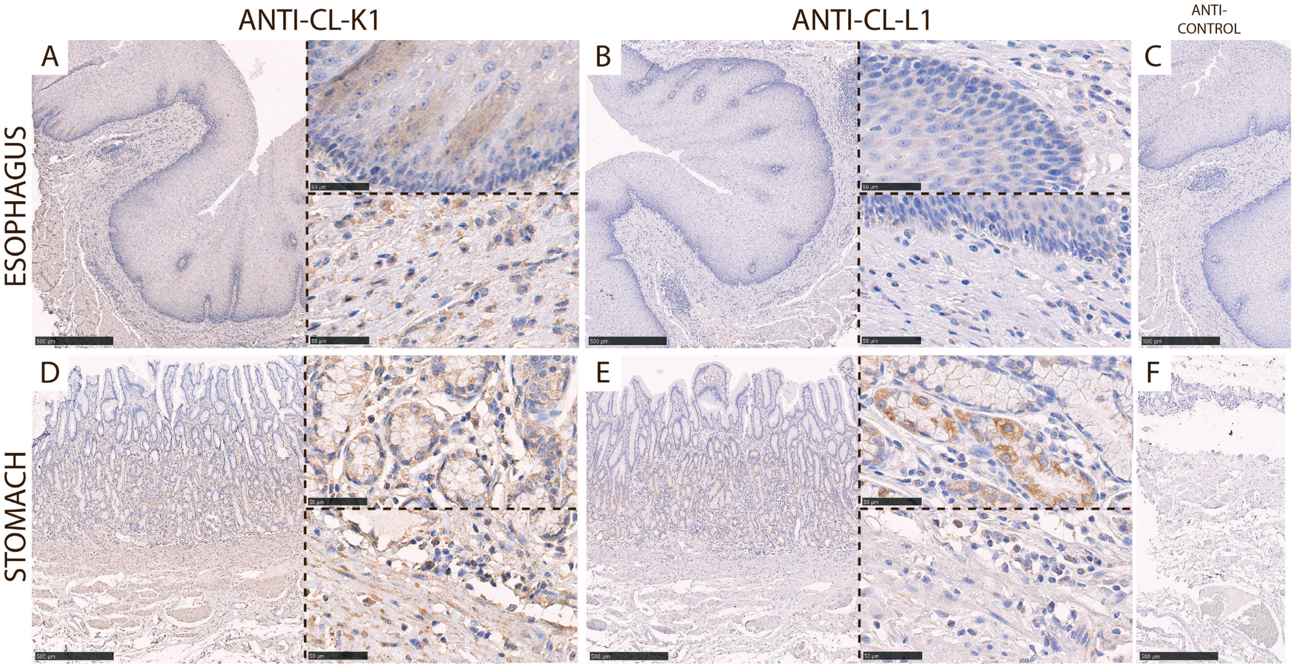

Suppl. figure 2. Immunohistochemical localization of CL-K1 and CL-L1 in formalin fixed and paraffin embedded sections of the esophagus (A, B) and stomach (D, E). Scale bars in large sections and in isotype control sections (C, F, G) correspond to 500 µm and in small sections.

In **the esophagus** CL-K1 immunoreactivity was associated with the stratified squamous epithelium, the lamina propria and the submucosa. Within the epithelial layer, the cells of the basal zone and especially the secreting ducts stained positive. In the lamina propria and submucosa staining was scattered and observed to be located both intracellulary and extracellulary. CL-L1 immunoreactivity in the esophagus was weak to absent. In the pylorus part of **the stomach,** gastric epithelial mucosal cells stained CL-K1 and CL-L1 immunoreactivity was mainly found in the epithelial cells at the narrow neck zone, situated between the superficial zone and substantial deep zone. Both the narrow neck zone and substantial deep zone are made of mucous secreting cells.

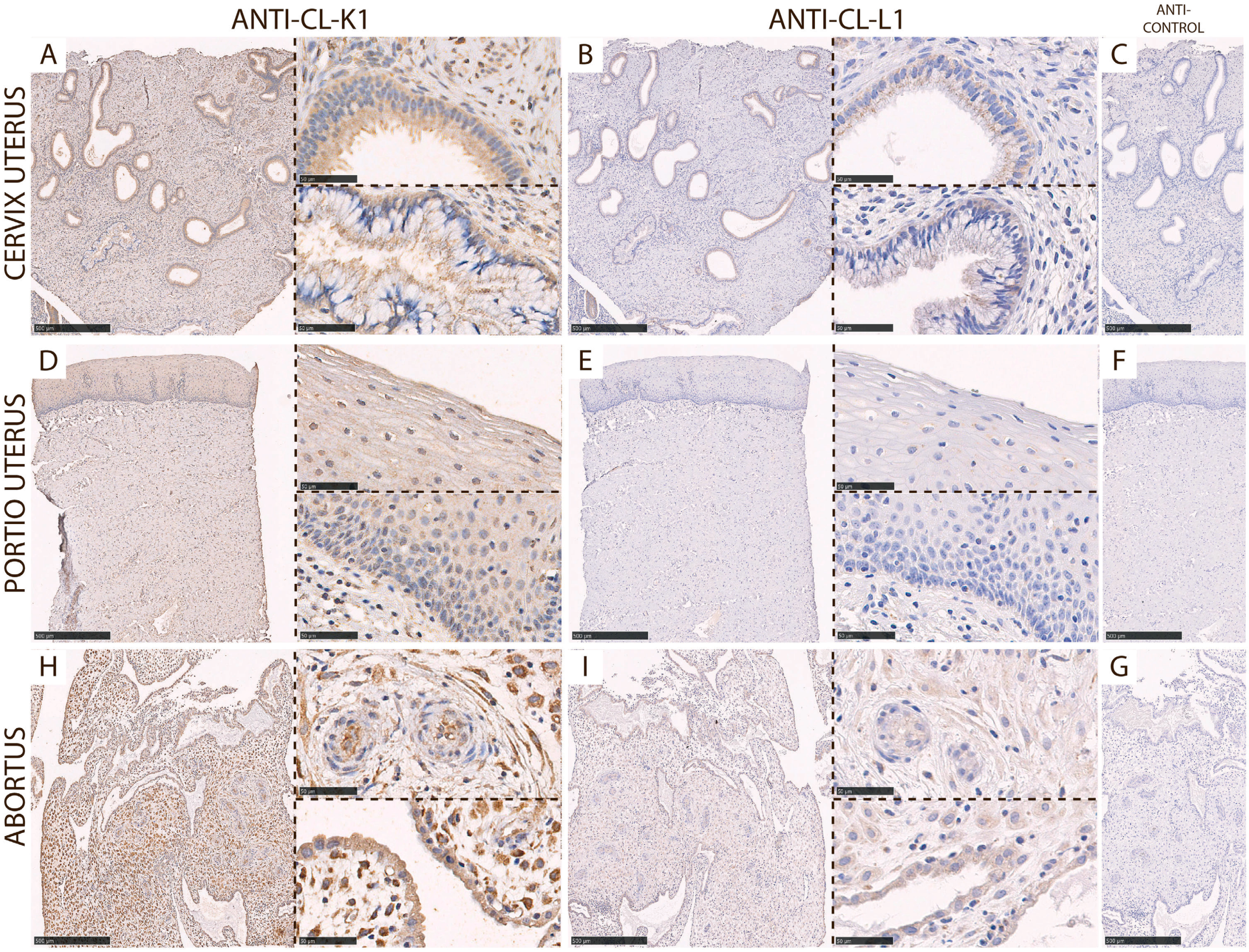

Suppl. figure 3. Immunohistochemical localization of CL-K1 and CL-L1 in formalin fixed and paraffin embedded sections of the cervix uterus (A, B), portio uterus (D, E) and abortus (H and I). Scale bars in large sections and in isotype control sections (C, F, G) correspond to 500 µm and in small sections. In the **cervix uterus** (endocervix), CL-K1 immunoreactivity was strongly associated with the simple columnar epithelial cells at the lumen of "cervical glands", representing cleft-like in-foldings of the surface epithelium. Further CL-K1 staining was associated with goblet cells in involuted glandular structures. CL-L1 immunoreactivity was weak in comparison with CL-K1 but present at the same localization. In **the portio uterus** (ecto and endocervix), moderate CL-K1 immunoreactivity was associated with stratified epithelium and basal cells in the ectocervix and weakly with stroma cells in the endocervix. In **the abortus** (abortive tissue), CL-K1 and CL-L1 immunoreactivity was associated with various structures, including tubules, blastema, glomeruli and spindle cell stroma.

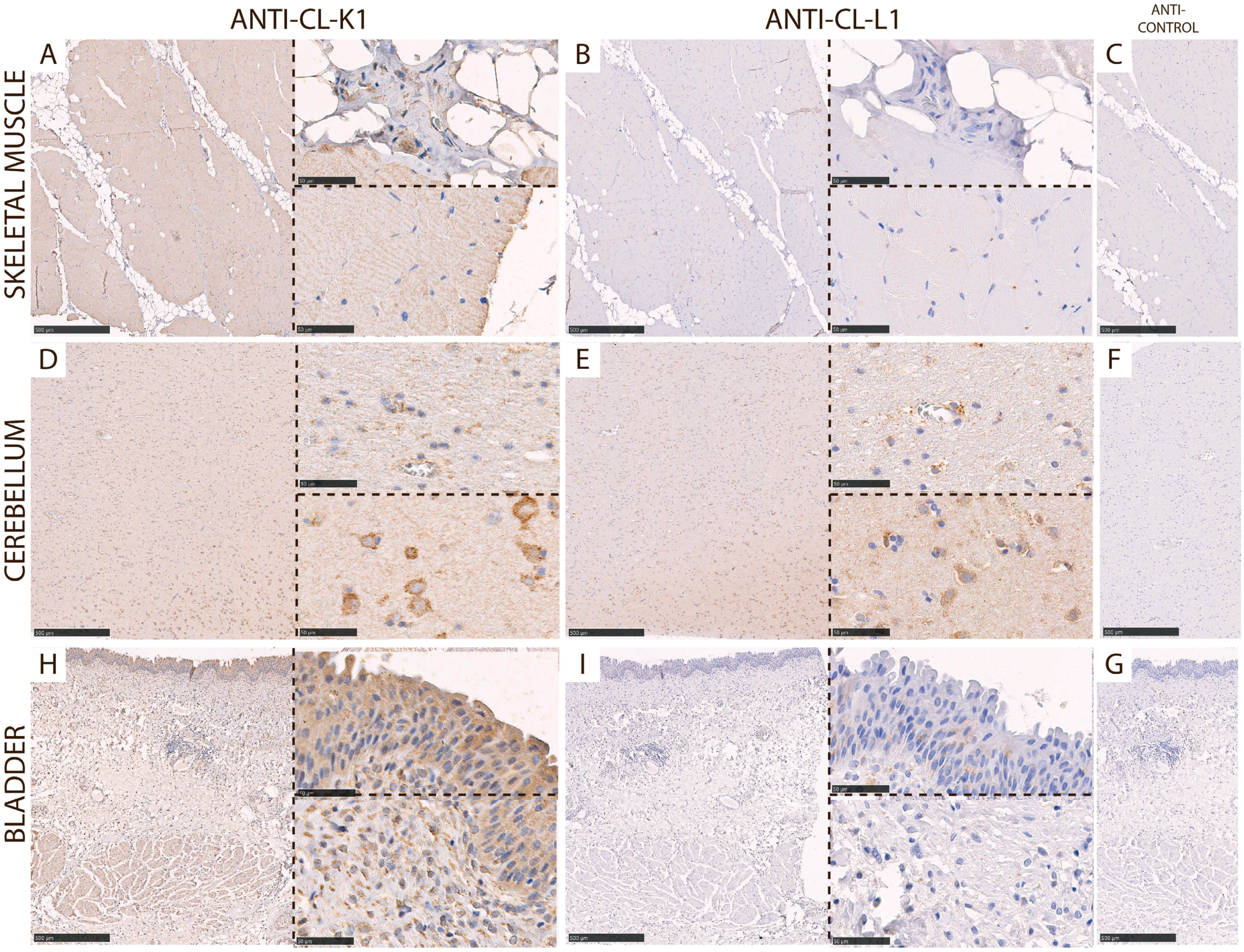

Suppl. figure 4. Immunohistochemical localization of CL-K1 and CL-L1 in formalin fixed and paraffin embedded sections of the skeletal muscle (A, B), cerebellum (D, E) and urinary bladder (H and I). Scale bars in large sections and in isotype control sections (C, F, G) correspond to 500 µm and in small sections.

In **the skeletal muscle**, weak to moderate CL-K1 immunoreactivity was mainly associated with muscle cells and infrequently with cells in the septa (connective tissue) surrounded by capillaries. Within the muscle cells, CL-K1 appeared to stain in a pattern that resembled the pattern of myosin, however, with some variation between separate cells. With the exception of some very scattered foci that could represent nerve cells, CL-L1 immunoreactivity was absent in the muscle.

In the cerebellum, CL-K1 and CL-L1 immunoreactivity was associated with scattered staining of cells in both strata (outer, deeper) of the molecular layer, with a tendency of pronounced staining cells in the surrounding of capillaries in the outer strata. In the deeper strata staining was especially associated with relative large cells with pale blue stained nuclei, which potential represent basket cells. In **the urinary bladder,** CL-K1 immunoreactivity was associated the multilayered epithelium compromising the urothelium at the luminal surface. All layers (basal, intermediate and umbrella cells) of the uroethelium stained equally positive for CL-K1. Weak to moderate CL-K1 staining of both the lamina propria and smooth muscle cells was also observed. CL-L1 staining was absent or weak and only localized to few cells of the urothelium.

SUPPLEMENTAL TABLE I

CL-L1 RNA	***HPA*** (TPM)	(norm. %)	***GTEx*** (RPKM)	(norm. %)	***Fantom5*** (TPM)	(norm. %)	***Average*** (norm. %)	SEM or DM	**Levels**
Adipose	0.0	0.0	0.0	0.0	0.0	0.0	**0.0**	0.0	**-**
Adrenal	0.3	0.6	0.3	2.9			**1.7**	1.2	**+**
Appendix	0.0	0.0			0.0	0.0	**0.0**	0.0	**-**
Bone marrow	0.0	0.0					**0.0**	0.0	**-**
Brain					0.0	0.0	**0.0**	0.0	**-**
Breast	0.1		0.0	0.0	0.0	0.0	**0.1**	0.1	**-**
Caudate			0.0	0.0	0.0	0.0	**0.0**	0.0	**-**
Cerebellum			0.0	0.0	0.0	0.0	**0.0**	0.0	**-**
Cerebral cortex	0.3	0.6	0.0	0.0			**0.3**	0.3	**+**
Cervix uterus	0.0	0.0	0.0	0.0	0.0	0.0	**0.0**	0.0	**-**
Colon	0.1	0.2	0.0	0.0	0.0	0.0	**0.1**	0.1	**-**
Duodenum	0.2	0.4					**0.4**	0.0	**+**
Endometrium	0.1	0.2	0.0	0.0	0.0	0.0	**0.1**	0.1	**-**
Epididymis	0.1	0.2			0.0	0.0	**0.1**	0.1	**-**
Esophagus	0.0	0.0	0.0	0.0	0.0	0.0	**0.0**	0.0	**-**
Fallopian tube	0.2	0.4	0.0	0.0			**0.2**	0.2	**+**
Gallbladder	1.6	3.2			1.4	3.5	**3.3**	0.2	**+**
Heart	0.4	0.8	0.1	1.0	0.0	0.0	**0.6**	0.3	**+**
Hippocampus			0.0	0.0	0.0	0.0	**0.0**	0.0	**-**
Hypothalamus			0.0	0.0			**0.0**	0.0	**-**
Kidney	0.6	1.2	0.2	1.9	0.0	0.0	**1.0**	0.6	**+**
Liver	51.1	100.0	10.4	100.0	40.0	100.0	**<u>100</u>**	-	**+++**
Lung	2.6	5.1	0.5	4.8	0.2	0.5	**3.5**	1.5	**+**
Lymph node	0.0	0.0			0.4	1.0	**0.5**	0.5	**+**
Ovary	0.0	0.0	0.0	0.0			**0.0**	0.0	**-**
Pancreas	0.0	0.0	0.0	0.0			**0.0**	0.0	**-**
Parathyroid	0.0	0.0					**0.0**	0.0	**-**
Pituary			0.1	1.0			**0.0**	0.0	**-**
Placenta	25.7	50.3	0.1	1.0	5.3	13.3	**21.6**	14.8	**+++**
Prostate	1.0	2.0	0.1	1.0	2.7	6.8	**3.2**	1.8	**+**
Rectum	0.1	0.2					**0.2**	0.0	**+**
Retina					0.0	0.0	**0.0**	0.0	**-**
Salivary gland	0.0	0.0	0.0	0.0	0.0	0.0	**0.0**	0.0	**-**
Seminal vesicle	0.4	0.8			0.0	0.0	**0.4**	0.4	**+**
Skel. muscle	0.0	0.0	0.0	0.0	0.0	0.0	**0.0**	0.0	**-**
Skin	0.0	0.0	0.0	0.0			**0.0**	0.0	**-**
Small intestine	0.2	0.4	0.0	0.0	0.7	1.75	**0.7**	0.5	**+**
Smooth muscle	0.0	0.0			0.0	0.0	**0.0**	0.0	**-**
Spleen	0.0	0.0	0.0	0.0	0.0	0.0	**0.0**	0.0	**-**
Stomach	0.1	0.2	0.0	0.0			**0.1**	0.1	**-**
Testis	0.1	0.2	0.0	0.0	0.0	0.0	**0.0**	0.1	**-**
Thyroid	4.9	9.6	0.3	2.9	0.0	0.0	**4.2**	2.8	**+**
Tonsil	0.0	0.0			0.0	0.0	**0.0**	0.0	**-**
Urinary bladder	0.4	0.8	0.0	0.0	0.0	0.0	**0.3**	0.3	**+**
Vagina			0.0	0.0	0.0	0.0	**0.0**	0.0	**-**

Suppl. table I. CL-L1 mRNA transcriptome data obtained from HPA (The Human Protein Atlas, Sweden), GTEx (Genotype-Tissue Expression project (NIH, USA) and FANTOM5 (RIKEN, Japan) given in TPM (transcripts per million) or RPKM (Reads Per Kilobase of transcript model per Million of mapped reads) and normalized in % to the level in the liver, defined as 100%. Variance is given as SEM (std. error of mean), when N = 3) or as DM (deviation from mean) when n < = 2. Levels are defined as ***absent*** (**-**: average < 0.1%), ***low*** (**+**: 0.1 ≤ average < 4.6%), ***medium*** (**+++**: 4.6 ≤ average < 21.5%) and ***high*** (**+++**: 21.5 ≤ average). The minimal discriminator in "absent" of 0.1% corresponds to 1‰ of the maximum value of averaged normalized levels (100%). The limits of 4.6 and 21.5% correspond to the 3rd root of 100% and the $3rd^2$, respectively.

SUPPLEMENTAL TABLE II

CL-K1 RNA	***HPA*** (TPM)	(norm. %)	***GTEx*** (RPKM)	(norm. %)	***Fantom5*** (TPM)	(norm. %)	***Average*** (norm. %)	SEM or DM	**Levels**
Adipose	4.6	13.5	3.7	11.4	1.5	02.5	**9.2**	3.4	**++**
Adrenal	36.3	106.8	16.3	50.3			**78.6**	28.2	**+++**
Appendix	1.6	4.7			0.0	0.0	**2.4**	2.4	**+**
Bone marrow	4.6	13.5					**13.5**	0.0	**++**
Brain					0.5	0.8	**0.8**	0.0	**+**
Breast	0.9	2.7	1.4	4.3	1.2	2.0	**3.0**	1.2	**+**
Caudate			0.5	1.5	0.0	0.0	**0.8**	0.8	**+**
Cerebellum			1.1	3.4	0.5	0.8	**2.1**	1.2	**+**
Cerebral cortex	1.6	4.7	0.4	1.2			**3.0**	1.7	**+**
Cervix uterus	1.4	4.1	0.6	1.9	0.0	0.0	**2.0**	1.2	**+**
Colon	1.6	4.7	1.0	3.1	1.9	3.2	**3.7**	0.9	**+**
Duodenum	4.3	12.7					**12.7**	0.0	**++**
Endometrium	2.0	5.9	0.5	1.5	0.9	1.5	**3.7**	2.2	**+**
Epididymis	1.3	3.8			0.8	1.3	**2.6**	1.2	**+**
Esophagus	1.9	5.6	0.5	1.5	0.9	1.5	**2.9**	1.6	**+**
Fallopian tube	2.9	8.5	2.5	7.7			**8.1**	0.4	**++**
Gallbladder	102.4	301.2			83.5	139.2	**220.2**	81.0	**+++**
Heart	11.2	33.0	7.4	22.8	4.9	8.2	**21.3**	7.2	**++**
Hippocampus			0.4	1.2	0.0	0.0	**0.6**	0.6	**+**
Hypothalamus			0.3	0.9			**0.9**	0.0	**+**
Kidney	18.3	53.8	8.0	24.7	3.1	5.2	**27.9**	14.1	**++**
Liver	34.0	100.0	32.4	100.0	60.0	100.0	**100**	-	**+++**
Lung	2.4	7.1	1.0	3.1	0.0	0.0	**3.4**	2.0	**+**
Lymph node	2.9	8.5			1.4	2.3	**5.4**	3.1	**+**
Ovary	30.0	88.2	21.4	66.0	8.3	12.8	**56.0**	22.1	**+++**
Pancreas	2.9	8.5	3.1	9.6	7.5	12.5	**10.2**	1.2	**++**
Parathyroid	2.7	7.9					**7.9**	0.0	**++**
Pituary			2.9	9.0	2.5	4.2	**6.6**	2.4	**++**
Placenta	4.7	13.8			3.0	5.0	**9.4**	4.1	**++**
Prostate	2.9	8.5	1.6	4.9	1.5	2.5	**5.3**	1.8	**+**
Rectum	1.6	4.7					**4.7**	0.0	**+**
Retina					0.9	1.5	**1.5**	0.0	**+**
Salivary gland	0.0	0.0	0.2	0.6	0.8	1.3	**0.7**	0.4	**+**
Seminal vesicle	2.9	8.5			0.0	0.0	**4.3**	4.3	**+**
Skel. muscle	0.0	0.0	0.0	0.0	0.7	1.2	**0.4**	0.4	**+**
Skin	2.9	8.5	0.0	0.0			**4.3**	4.3	**+**
Small intestine	5.4	15.9	1.3	4.1	4.2	7.0	**9.0**	1.6	**++**
Smooth muscle	2.0	5.9			1.2	2.0	**3.9**	1.9	**+**
Spleen	5.2	15.3	1.6	4.9	1.2	2.0	**7.4**	4.0	**++**
Stomach	2.4	7.1	1.1	3.4			**5.2**	1.8	**+**
Testis	11.7	34.4	4.6	14.2	4.1	6.8	**18.5**	8.2	**++**
Thyroid	11.0	32.4	7.9	24.4	7.7	12.8	**23.2**	5.7	**++**
Tonsil	0.8	2.4			0.0	0.0	**1.2**	1.2	**+**
Urinary bladder	4.5	13.2	2.4	7.4	0.9	1.5	**7.4**	3.4	**++**
Vagina			0.5	1.5	1.2	2.0	**1.8**	0.2	**+**

Suppl. table II. CL-K1 mRNA transcriptome data obtained from HPA (The Human Protein Atlas, Sweden), GTEx (Genotype-Tissue Expression project (NIH, USA) and FANTOM5 (RIKEN, Japan) given in TPM (transcripts per million) or RPKM (Reads Per Kilobase of transcript model per Million of mapped reads) and normalized in % to the level in the liver, defined as 100%. Variance is given as SEM, when N = 3) or as DM (deviation from mean) when n < 3. Levels are defined as ***absent*** (**-**: average < 0.2%), ***low*** (**+**: 0.2 ≤ average < 6.0%), ***medium*** (**++**: 6.0 ≤ average < 36.4%) and ***high*** (**+++**: 36.4 ≤ average). The minimal discriminator in "absent" of 0.2% corresponds to 1‰ of the maximum value of averaged normalized levels (220.2%). The limits of 6.0 and 36.4% correspond to the 3rd root of 220.2% and the $3rd^2$, respectively.

+SUPPLEMENTAL TABLE III

MBL RNA	***HPA***		***GTEx***		***Fantom5***		***Average***	SEM or	**Levels**
	(TPM)	(norm. %)	(RPKM)	(norm. %)	(TPM)	(norm. %)	(norm. %)	DM	
Adipose	0.0	0.0	0.0	0.0	0.0	0.0	**0.0**	0.0	-
Adrenal	0.0	0.0	0.0	0.0			**0.0**	0.0	-
Appendix	0.0	0.0			0.0	0.0	**0.0**	0.0	-
Bone marrow	0.0	0.0				0.0	**0.0**	0.0	-
Brain					0.0	0.8	**0.0**	0.0	-
Breast	0.0	0.0	0.0	0.0			**0.0**	0.0	-
Caudate			0.0	0.0	0.0	0.0	**0.0**	0.0	-
Cerebellum			0.0	0.0	0.0	0.0	**0.0**	0.0	-
Cerebral cortex	0.0	0.0	0.0	0.0			**0.0**	0.0	-
Cervix uterus	0.0	0.0	0.0	0.0	0.0	0.0	**0.0**	0.0	-
Colon	0.0	0.0	0.0	0.0	0.0	0.0	**0.0**	0.0	-
Duodenum	0.0	0.0					**0.0**	0.0	-
Endometrium	0.0	0.0	0.0	0.0	0.0	0.0	**0.0**	0.0	-
Epididymis	0.0	0.0			0.8	1.3	**0.0**	0.0	-
Esophagus	0.0	0.0	0.0	0.0			**0.0**	0.0	-
Fallopian tube	0.0	0.0	0.0	0.0	0.0	0.0	**0.0**	0.0	-
Gallbladder	0.0	0.0					**0.0**	0.0	-
Heart	0.0	0.0	0.0	0.0			**0.0**	0.0	-
Hippocampus			0.0	0.0	0.0	0.0	**0.0**	0.0	-
Hypothalamus					0.0	0.0	**0.0**	0.0	-
Kidney	0.0	0.0	0.0	0.0	0.0	0.0	**0.0**	0.0	-
Liver	141.3	100.0	43.7	100.0	61.9	100.0	**<u>100</u>**	-	**+++**
Lung	0.0	0.0	0.0	0.0	0.0	0.0	**0.0**	0.0	-
Lymph node	0.0	0.0					**0.0**	0.0	-
Ovary	0.0	0.0	0.0	0.0	0.0	0.0	**0.0**	0.0	-
Pancreas	0.0	0.0	0.0	0.0	0.0	0.0	**0.0**	0.0	-
Parathyroid	0.0	0.0					**0.0**	0.0	-
Pituary			0.0	0.0	0.0	0.0	**0.0**	0.0	-
Placenta	0.0	0.0					**0.0**	0.0	-
Prostate	0.0	0.0	0.0	0.0			**0.0**	0.0	-
Rectum	0.0	0.0					**0.0**	0.0	-
Retina					0.0	0.0	**0.0**	0.0	-
Salivary gland	0.0	0.0	0.0	0.0	0.0	0.0	**0.0**	0.0	-
Seminal vesicle	0.0	0.0			0.0	0.0	**0.0**	0.0	-
Skel. muscle	0.0	0.0	0.0	0.0	0.0	0.0	**0.0**	0.0	-
Skin	0.0	0.0	0.0	0.0			**0.0**	0.0	-
Small intestine	0.0	0.0	0.0	0.0	0.0	0.0	**0.0**	0.0	-
Smooth muscle	0.0	0.0			0.0	0.0	**0.0**	0.0	-
Spleen	0.0	0.0	0.0	0.0	0.0	0.0	**0.0**	0.0	-
Stomach	0.0	0.0	0.0	0.0			**0.0**	0.0	-
Testis	0.0	0.0	0.0	0.0	0.0	0.0	**0.0**	0.0	-
Thyroid	0.0	0.0	0.0	0.0	0.0	0.0	**0.0**	0.0	-
Tonsil	0.0	0.0			0.0	0.0	**0.0**	0.0	-
Urinary bladder	0.0	0.0	0.0	0.0	0.0	0.0	**0.0**	0.0	-
Vagina			0.0	0.0	0.0	0.0	**0.0**	0.0	-

Suppl. table III. MBL mRNA transcriptome data obtained from HPA (The Human Protein Atlas, Sweden), GTEx (Genotype-Tissue Expression project (NIH, USA) and FANTOM5 (RIKEN, Japan) given in TPM (transcripts per million) or RPKM (Reads Per Kilobase of transcript model per Million of mapped reads) and normalized in % to the level in the liver, defined as 100%. Variance is given as SEM, when N = 3) or as DM (deviation from mean) when n < 3. Levels are defined as ***absent*** (**-**: average < 0.1%), ***low*** (**+**: 0.1 ≤ average < 4.6%), ***medium*** (**++**: 4.6 ≤ average < 21.5%) and ***high*** (***+++** 21.5 ≤ average). The minimal discriminator in "absent" of 0.1% corresponds to 1‰ of the maximum value of averaged normalized levels (100%). The limits of 4.6 and 21.5% correspond to the 3rd root of 100% and the $3rd^2$, respectively.

SUPPLEMENTAL TABLE IV

MASP-1 mRNA	***Seyfarth et al. 2006*** *(norm. %)*	***Degn et al. 2009*** *(norm. %)*	***Average*** *(norm. %)*	Variance (DM)	**Levels**
Adipose		0	**0**	0	**-**
Brain	10	2	**6**	4	**++**
Cervix uterus		4	**4**	0	**+**
Colon	16	0	**8**	8	**++**
Esophagus		0	**0**	0	**-**
Heart	10	0	**5**	5	**++**
Kidney	30	0	**15**	15	**++**
Liver	100	100	**100**	-	**+++**
Lung	18	0	**9**	9	**++**
Ovary	0	0	**0**	0	**-**
Pancreas	0		**0**	0	**-**
Placenta	16	0	**8**	0	**-**
Prostate	0	0	**0**	0	**-**
Skel. muscle	0	0	**0**	0	**-**
Small intestine	39	0	**20**	20	**++**
Spleen	0	0	**0**	0	**-**
Testis	0	0	**0**	0	**-**
Thymus	0	0	**0**	0	**-**
Thyroid		0	**0**	0	**-**
Trachea		0	**0**	0	**-**
Urinary bladder		0	**0**	0	**-**

Suppl. table IV. MASP-1 mRNA expression data obtained from Seyfarth et al. 2006 (30) and Degn et al. 2009 (32) normalized in % to the level in the liver, defined as 100%. Variance is given as DM (deviation from mean). Levels are defined as ***absent*** (**-**: average < 0.1%), ***low*** (**+**: 0.1 ≤ average < 4.6%), ***medium*** (**++**: 4.6 ≤ average < 21.5%) and ***high*** (**+++**: 21.5 ≤ average). The minimal discriminator in "absent" of 0.1% corresponds to 1‰ of the maximum value of averaged normalized levels (100%). The limits of 4.6 and 21.5% correspond to the 3rd root of 100% and the 3rd^{2}, respectively.

SUPPLEMENTAL TABLE V

MASP-3 mRNA	***Seyfarth et al. 2006*** *(norm. %)*	***Degn et al. 2009*** *(norm. %)*	***Average*** *(norm. %)*	Variance (DM)	**Levels**
Adipose		2	**2**	0	**+**
Brain	41	24	**33**	9	**++**
Cervix uterus		68	**68**	0	**+++**
Colon	88	20	**54**	34	**+++**
Esophagus		2	**2**	0	**+**
Heart	76	4	**40**	36	**+++**
Kidney	33	0	**17**	17	**++**
Liver	100	100	**100**	-	**+++**
Lung	59	6	**33**	27	**+++**
Ovary	59	6	**33**	27	**+++**
Pancreas	34		**34**	0	**+++**
Placenta	51	18	**34**	16	**+++**
Prostate	73	20	**47**	27	**+++**
Skel. muscle	74	0	**37**	37	**+++**
Small intestine	57	0	**28**	80	**+++**
Spleen	40	0	**20**	20	**++**
Testis	31	8	**19**	11	**++**
Thymus	23	0	**12**	12	**++**
Thyroid		0	**0**	0	**-**
Trachea		0	**0**	0	**-**
Urinary bladder		16	**16**	0	**++**

Suppl. table V. MASP-3 mRNA expression data obtained from Seyfarth et al. 2006 (30) and Degn et al. 2009 (32) normalized in % to the level in the liver, defined as 100%. Variance is given as DM (deviation from mean). Levels are defined as ***absent*** (**-**: average < 0.1%), ***low*** (+: 0.1 ≤ average < 4.6%), ***medium*** (**++**: 4.6 ≤ average < 21.5%) and ***high*** (**+++**: 21.5 ≤ average). The minimal discriminator in "absent" of 0.1% corresponds to 1‰ of the maximum value of averaged normalized levels (100%). The limits of 4.6 and 21.5% correspond to the 3rd root of 100% and the 3rd^{2}, respectively.

SUPPLEMENTAL TABLE VI

MAp44 mRNA	***Skjoedt et al. 2010*** *(norm. %)*	***Degn et al. 2009*** *(norm. %)*	***Average*** *(norm. %)*	Variance (DM)	**Levels**
Adipose		0	**0**	0	**-**
Brain	4	8	**6**	2	**++**
Cervix uterus		6	**6**	0	**++**
Colon	0	2	**1**	1	**+**
Esophagus		0	**0**	0	**-**
Heart	100	100	**100**	-	**+++**
Kidney		0	**0**	0	**-**
Liver	0	10	**5**	5	**++**
Lung	30	0	**15**	15	**++**
Ovary		0	**0**	0	**-**
Pancreas	0		**0**	0	**-**
Placenta	0	0	**0**	0	**-**
Prostate	0	0	**0**	0	**-**
Skel. muscle	61	0	**30**	30	**+++**
Small intestine		0	**0**	0	**-**
Spleen	0	0	**0**	0	**-**
Testis		0	**0**	0	**-**
Thymus		0	**0**	o	**-**
Thyroid		0	**0**	0	**-**
Trachea		0	**0**	0	**-**
Urinary bladder		0	**0**	0	**-**

Suppl. table VI. MAp44 mRNA expression data obtained from Skjoedt et al. 2010 (33) and Degn et al. 2009 (32) normalized in % to the level in the heart, defined as 100%. Variance is given as DM (deviation from mean). Levels are defined as ***absent*** (**-**: average < 0.1%), ***low*** (**+**: 0.1 ≤ average < 4.6%), ***medium*** (**++**: 4.6 ≤ average < 21.5%) and ***high*** (**+++**: 21.5 ≤ average). The minimal discriminator in "absent" of 0.1% corresponds to 1‰ of the maximum value of averaged normalized levels (100%). The limits of 4.6 and 21.5% correspond to the 3rd root of 100% and the $3rd^2$, respectively.

SUPPLEMENTAL TABLE VII

MASP-2 mRNA	***Seyfarth et al. 2006*** *(norm. %)*	***Degn et al. 2011*** *(norm. %)*	***Average*** *(norm. %)*	Variance (DM)	**Levels**
Adipose		0	**0**	0	**-**
Brain	0	0	**0**	0	**-**
Cervix uterus		0	**0**	0	**-**
Colon	0	0	**0**	0	**-**
Esophagus		0	**0**	0	**-**
Heart	0	0	**0**	0	**-**
Kidney	0	0	**0**	0	**-**
Liver	100	100	**100**	-	**+++**
Lung	0	0	**0**	0	**-**
Ovary	0	0	**0**	0	**-**
Pancreas	0		**0**	0	**-**
Placenta	0	0	**0**	0	**-**
Prostate	0	0	**0**	0	**-**
Skel. muscle	0	0	**0**	0	**-**
Small intestine	34	0	**17**	17	**++**
Spleen	0	0	**0**	0	**-**
Testis	10	0	**5**	5	**++**
Thymus	0	0	**0**	o	**-**
Thyroid		0	**0**	0	**-**
Trachea		0	**0**	0	**-**
Urinary bladder		0	**0**	0	**-**

Suppl. table VII. MASP-2 mRNA expression data obtained from Seyfarth et al. 2006 (30) and Degn et al. 2011 (31) normalized in % to the level in the liver, defined as 100%. Variance is given as DM (deviation from mean). Levels are defined as ***absent*** (**-**: average < 0.1%), ***low*** (**+**: 0.1 ≤ average < 4.6%), ***medium*** (**++**: 4.6 ≤ average < 21.5%) and ***high*** (**+++**: 21.5 ≤ average). The minimal discriminator in "absent" of 0.1% corresponds to 1‰ of the maximum value of averaged normalized levels (100%). The limits of 4.6 and 21.5% correspond to the 3rd root of 100% and the 3rd^2, respectively.

SUPPLEMENTAL TABLE IIX

MAp19 mRNA	***Degn et al. 2011*** *(norm. %)*	**Levels**
Adipose	0	-
Brain	0	-
Cervix uterus	0	-
Colon	0	-
Esophagus	0	-
Heart	0	-
Kidney	16	++
Liver	100	+++
Lung	0	-
Ovary	0	-
Placenta	0	-
Prostate	0	-
Skel. muscle	0	-
Small intestine	0	-
Spleen	0	-
Testis	0	-
Thymus	0	-
Thyroid	30	+++
Trachea	0	-
Urinary bladder	0	-

Suppl. table IIX. MASP-2 mRNA expression data obtained from Degn et al. 2011 (31) normalized in % to the level in the liver, defined as 100%. Levels are defined as ***absent*** (**-**: average < 0.1%), ***low*** (**+**: 0.1 ≤ average < 4.6%), ***medium*** (**++**: 4.6 ≤ average < 21.5%) and ***high*** (**+++**: 21.5 ≤ average). The minimal discriminator in "absent" of 0.1% corresponds to 1‰ of the maximum value of averaged normalized levels (100%). The limits of 4.6 and 21.5% correspond to the 3rd root of 100% and the $3rd^2$, respectively.
